# Conduction System Pacing for Post Transcatheter Aortic Valve Replacement Patients: Comparison With Right Ventricular Pacing

**DOI:** 10.3389/fcvm.2021.772548

**Published:** 2021-11-30

**Authors:** Hong-Xia Niu, Xi Liu, Min Gu, Xuhua Chen, Chi Cai, Minsi Cai, Shu Zhang, Wei Hua

**Affiliations:** Cardiac Arrhythmia Center, Fuwai Hospital, National Center for Cardiovascular Diseases, Chinese Academy of Medical Sciences and Peking Union Medical College, Beijing, China

**Keywords:** conduction system pacing, transcatheter aortic valve replacement, his bundle pacing, left bundle branch pacing, right ventricular pacing, outcomes

## Abstract

**Introduction:** For patients who develop atrioventricular block (AVB) following transcatheter aortic valve replacement (TAVR), right ventricular pacing (RVP) may be associated with adverse outcomes. We assessed the feasibility of conduction system pacing (CSP) in patients who developed AVB following TAVR and compared the procedural and clinical outcomes with RVP.

**Methods:** Consecutive patients who developed AVB following TAVR were prospectively enrolled, and were implanted with RVP or CSP. Procedural and clinical outcomes were compared among different pacing modalities.

**Results:** A total of 60 patients were enrolled, including 10 who were implanted with His bundle pacing (HBP), 20 with left bundle branch pacing (LBBP), and 30 with RVP. The HBP group had significantly lower implant success rate, higher capture threshold, and lower R-wave amplitude than the LBBP and RVP groups (*p* < 0.01, respectively). The RVP group had a significantly longer paced QRS duration (153.5 ± 6.8 ms, *p* < 0.01) than the other two groups (HBP: 121.8 ± 8.6 ms; LBBP: 120.2 ± 10.6 ms). During a mean follow-up of 15.0 ± 9.1 months, the LBBP group had significantly higher left ventricular ejection fraction (LVEF) (54.9 ± 6.7% vs. 48.9 ± 9.1%, *p* < 0.05) and shorter left ventricular end-diastolic diameter (LVEDD) (49.7 ± 5.6 mm vs. 55.0 ± 7.7 mm, *p* < 0.05) than the RVP group. While the HBP group showed trends of higher LVEF (*p* = 0.016) and shorter LVEDD (*p* = 0.017) than the RVP group. Four patients in the RVP group died—three deaths were due to progressive heart failure and one was due to non-cardiac reasons. One death in the LBBP group was due to the non-cardiac reasons.

**Conclusions:** CSP achieved shorter paced QRS duration and better cardiac structure and function in post-TAVR patients than RVP. LBBP had a higher implant success rate and better pacing parameters than HBP.

## Introduction

Transcatheter aortic valve replacement (TAVR) is an effective treatment option for patients with severe aortic stenosis at moderate-to-high surgical risk. However, a high-degree or complete atrioventricular block (AVB) is a well-recognized complication of TAVR, which requires permanent pacemaker implantation. Patients undergoing TAVR usually have left ventricular systolic dysfunction, and right ventricular pacing (RVP) in these patients may increase the risk of heart failure (HF) and is associated with adverse clinical outcomes (1). His bundle pacing (HBP), which is regarded as a physiological pacing modality, is associated with reduced risk of HF hospitalization and pacing-induced cardiomyopathy compared with RVP (2). However, the implant success rate of HBP in post-TAVR patients is only about 50–63% (3, 4). More recently, left bundle branch pacing (LBBP) has been shown to be a safe and effective alternative to HBP, and is considered an alternative approach for conduction system pacing (CSP) (5). Unlike HBP, LBBP is more likely to cross the block site and achieve ideal pacing parameters. Several small-sample studies have evaluated the feasibility of CSP in post-TAVR patients. However, comparisons between CSP and RVP in post-TAVR patients have not been well-described (3, 6, 7). In this study, we assessed the feasibility of CSP in a cohort of post-TAVR patients and compared the procedural and clinical outcomes of TAVR with RVP.

## Materials and Methods

**Figure 1:**
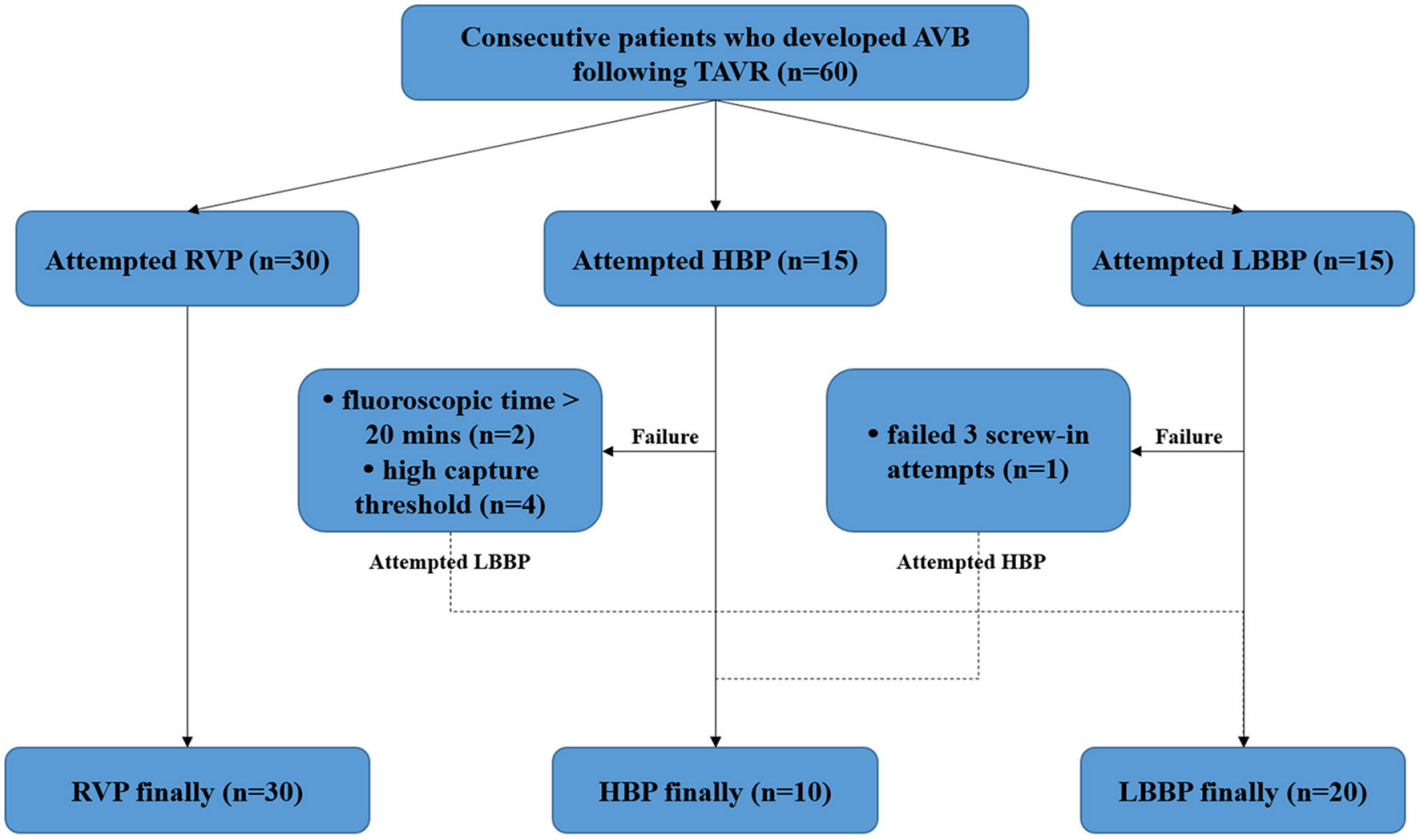
Study flow diagram. AVB, atrioventricular block; HBP, His bundle pacing; LBBP, left bundle branch pacing; RVP, right ventricular pacing; TAVR, transcatheter aortic valve replacement.

In this prospective, non-randomized, single-center study, 60 consecutive patients who developed AVB following TAVR and required pacemaker implantation were enrolled. Patients were excluded if they previously implanted with any cardiac implantable electronic devices. The patients were randomized to receive RVP or CSP. Those randomized to receive CSP were alternately allocated to attempt HBP or LBBP (first attempt HBP, second attempt LBBP, third attempt HBP, fourth attempt LBBP, etc…). However, if HBP was unsuccessful, LBBP was attempted and vice versa. If both types of the CSP (HBP and LBBP) were unsuccessful, RVP was finally performed (Figure 1). This study was approved by the Ethics Committee of Fuwai Hospital, and written informed consent was obtained from all patients.

### Implantation Procedure

In this study, TAVR was performed using the self-expandable Venus A-Valve (Venus MedTech, Hangzhou, China) in patients with symptomatic severe aortic stenosis at moderate-to-high surgical risk. The RVP implantation was performed using the conventional transvenous approach, and the ventricular lead was placed at the right ventricle. The implantation procedures of HBP and LBBP were performed using the conventional method or with the guidance of the visualization technique previously described by our team (8–10). All CSP implantations were performed using the fixed-curve C315 HIS sheath (Medtronic Inc, Minneapolis, MN) and the Select Secure 3830, pacing lead (Medtronic Inc, Minneapolis, MN).

### Target His Bundle Region

The His bundle (HB) region was defined as the region where the HB potential could be recorded or the HB could be captured by unipolar pacing. The HB region could also be located under the guidance of our visualization technique (8), which showed the location of the tricuspid value annulus (TVA) by injecting 10–20 mL contrast medium through the C315 HIS sheath below the root of the tricuspid septal leaflet. The fluoroscopic image of the TVA location was saved as an anatomic reference, which was then used to locate the HB region based on the positional relationship between the HB region and TVA as previously described (8).

### Target Left Bundle Branch Region

After the HB region was identified, the lead was moved toward the right ventricular apex by ~1–2 cm. The initial screw-in site was defined as right-side site of the ventricular septum where the paced QRS morphology in lead V1 showed a “W” pattern. Then, the lead was screwed deep into the myocardium by carefully monitoring the pacing morphology to confirm left bundle branch (LBB) capture. Successful LBB capture was assumed in patients with a paced QRS morphology in lead V1 showing a right bundle branch block (RBBB) pattern and met at least one of the three criteria including: (1) recording of an LBB potential; (2) short and constant left ventricular activation time (LVAT) at different pacing outputs or abruptly shortened LVAT at high output; and (3) demonstration of selective LBB capture. In addition, the target LBB region could also be located based on the positional relationship between the LBB region and TVA provided by the visualization technique (9).

### Lead Fixation and Testing

**Figure 2:**
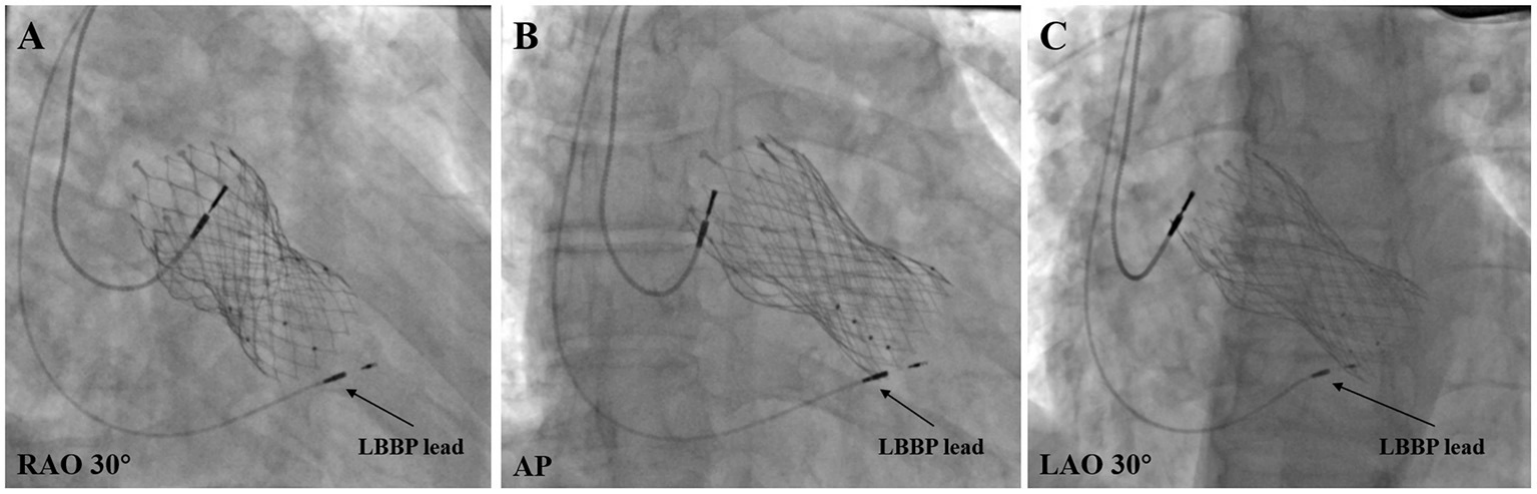
Final LBBP lead location in post-TAVR patients. **(A)** LBBP lead location under RAO 30° fluoroscopic view. **(B)** LBBP lead location under anteroposterior (AP) fluoroscopic view. **(C)** LBBP Lead location under LAO 30° fluoroscopic view. LBBP, left bundle branch pacing; LAO, left anterior oblique; RAO, right anterior oblique.

The lead was fixed at the ideal location where the pacing parameters were satisfactory (Figure 2). The CSP was considered unsuccessful if the capture threshold was >2.5 V/0.4 ms in three attempts, or the total fluoroscopic time was >20 min.

### Data Collection and Follow-Up

Data on baseline characteristics, valve types, and indications for pacemaker implantation were collected at enrollment. Post-implantation follow-up was performed at 3, 6, 12, and then routinely every 12 months. Data from the last follow-up with a minimal of 6 months were used for analysis. Echocardiographic measurements including left ventricular end-diastolic diameter (LVEDD) and left ventricular ejection fraction (LVEF) were recorded at baseline and during each follow-up visit. Pacing parameters including capture threshold, R-wave amplitude, and impedance were recorded during the procedure and during each follow-up visit. Procedure-related complications including capture threshold increase by >1 V/0.4 ms, loss of capture, lead septal perforation, and lead dislodgement were recorded during follow-up. Clinical endpoints including death or hospitalization for HF after pacemaker implantation were also evaluated during follow-up.

### Statistical Analysis

Continuous variables were presented as mean ± standard deviation, and categorical variables were expressed as frequencies or percentages. Analysis of variance (ANOVA) was used for multiple comparisons in normally distributed data among groups, and *post-hoc* tests were performed for variables that showed a statistically significant difference. Kruskal–Wallis test was performed for data that were not normally distributed. The chi-squared or Fisher' s exact tests were used for categorical variables to determine differences among groups. A two-sided *P*-value < 0.05 was considered to indicate statistically significant differences. All statistical analyses were performed using the SPSS Statistics version 22.0 (IBM Corporation, Armonk, NY).

## Results

### Baseline Characteristics

From April 2018 to December 2020, a total of 60 patients who developed AVB following TAVR and eventually had a pacemaker implanted in our center were prospectively enrolled. Baseline characteristics are summarized in Table 1. Briefly, patients' mean age was 78.2 ± 5.4 years, and 39 of 60 (65.0%) patients were male. Thirty-one (51.7%) patients had hypertension, 19 (31.7%) had diabetes mellitus, and 13 (21.7%) had coronary artery disease. For the baseline electrocardiogram, the mean native QRS duration was 134.1 ± 30.8 ms. Thirty-six (60%) patients had pre-existing conduction system block including LBBB in 13 (21.7%) and RBBB in 23 (38.3%). The mean LVEF was 52.1 ± 8.4 %, and the mean LVEDD was 52.4 ± 7.8 mm. All enrolled patients underwent TAVR using the self-expandable Venus A-Valve. The pacing indications included high-degree AVB and complete AVB, which accounted for 20 (33.3%) and 40 (66.7%) of the total patients, respectively.

**Table 1:** Baseline characteristics.

	**Value**
Number of patients	60 (100%)
**Demographics**	
Age (years)	78.2 ± 5.4
Male	39 (65.0%)
**Comorbidities**	
Hypertension	31 (51.7%)
Diabetes mellitus	19 (31.7%)
Coronary artery disease	13 (21.7%)
**Baseline electrocardiogram**	
QRS duration (ms)	134.1 ± 30.8
LBBB	13 (21.7%)
RBBB	23 (38.3%)
**Baseline echocardiography**	
LVEF (%)	52.1 ± 8.4
LVEDD (mm)	52.4 ± 7.8
**Valve type**	
Venus A-Valve	60 (100%)
**Indications for pacing**	
High-degree AVB	20 (33.3%)
Complete AVB	40 (66.7%)
Dual chamber pacemaker	56 (93.3%)
Conduction system pacing	30 (50%)
Follow-up duration (month)	15.0 ± 9.1

*AVB, atrioventricular block; LBBB, left bundle branch block; LVEDD, left ventricular end-diastolic diameter; LVEF, left ventricular ejection fraction; RBBB, right bundle branch block*.

### Procedural Outcomes

The procedural outcomes are shown in Figure 1. RVP was attempted in 30 patients, and all were successfully implanted. Of the 30 patients who underwent CSP, 15 patients first tried HBP, and nine patients had a successful outcome. In two patients, the fluoroscopic time was >20 min and in four patients, the capture threshold was high. Subsequently, LBBP was attempted and successfully performed on these six patients. LBBP was first tried in 15 patients and was successfully achieved in 14 patients. The procedure in the remaining one patient was considered unsuccessful because of the failure to screw the lead into the myocardium after three screw-in attempts; HBP was then performed in this patient. Finally, a total of 10 patients were assigned to the HBP group, 20 to the LBBP group, and 30 to the RVP group. As shown in Table 2, no significant differences were observed in baseline characteristics including electrocardiographic measurements, echocardiogram, New York Heart Association (NYHA) functional class and medical therapy among the three groups.

**Table 2:** Baseline characteristics among groups.

	**HBP group (*n* = 10)**	**LBBP group (*n* = 20)**	**RVP group (*n* = 30)**	***P*-value**
**Baseline electrocardiogram**				
QRS duration (ms)	132.2 ± 30.5	133.8 ± 32.9	134.9 ± 30.6	0.98
LBBB	2 (20.0%)	4 (20.0%)	7 (23.3%)	0.95
RBBB	4 (40.0%)	7 (35.0%)	12 (40.0%)	0.93
**NYHA functional class**				
II	1 (10.0%)	3 (15.0%)	4 (13.3%)	0.93
III	4 (40.0%)	8 (40.0%)	10 (33.3%)	0.87
IV	5 (50.0%)	9 (45.0%)	16 (53.3%)	0.85
**Baseline echocardiography**				
LVEF (%)	52.1 ± 5.3	51.9 ± 8.5	52.3 ± 9.3	0.98
LVEDD (mm)	53.3 ± 5.9	52.7 ± 8.1	51.8 ± 8.4	0.77
**Medications**				
ACEI/ARB	2 (20.0%)	4 (20.0%)	5 (16.7%)	0.95
Beta-blocker	2 (20.0%)	5 (25.0%)	5 (16.7%)	0.77
Diuretics	7 (70.0%)	12 (60.0%)	19 (63.3%)	0.86
Aldosterone antagonist	1 (10.0%)	2 (10.0%)	4 (13.3%)	0.92

*ACEI, angiotensin converting enzyme inhibitor; ARB, angiotensin receptor blocker; HBP, His bundle pacing; LBBB, left bundle branch block; LBBP, left bundle branch pacing; LVEDD, left ventricular end-diastolic diameter; LVEF, left ventricular ejection fraction; NYHA, New York Heart Association; RBBB, right bundle branch block; RVP, right ventricular pacing*.

As shown in Table 3, the implant success rate in the HBP group was significantly lower than that in the LBBP and RVP groups (62.5 vs. 95.2% vs. 100.0%, *p* < 0.01). No significant differences were observed in the paced QRS duration between the HBP and LBBP groups (121.8 ± 8.6 ms and 120.2 ± 10.6 ms), while in the RVP group, the paced QRS duration was significantly longer (153.5 ± 6.8 ms, *p* < 0.01). During the procedure, the capture threshold was significantly different among the three groups. Patients in the HBP group had the highest capture threshold (1.5 ± 0.4 V/0.4 ms), patients in the RVP group had the lowest (0.6 ± 0.2 V/0.4 ms), and those in the LBBP group had moderate capture threshold (0.8 ± 0.2 V/0.4 ms). The R-wave amplitude in the HBP group (5.7 ± 2.6 mV, *p* < 0.01) was significantly lower than those in the LBBP and RVP groups (11.2 ± 3.0 mV and 11.5 ± 3.4 mV). No significant differences were observed in the impedance among the three groups during the procedure (*p* = 0.76).

**Table 3:** Procedural and clinical outcomes among groups.

	**HBP group (*n* = 10)**	**LBBP group (*n* = 20)**	**RVP group (*n* = 30)**	***P*-value**
Implant success rate (%)	10/16 (62.5%)	20/21 (95.2%)	30/30 (100.0%)	<0.01^*^
Paced QRS duration (ms)	121.8 ± 8.6	120.2 ± 10.6	153.5 ± 6.8	<0.01^†^
Pacing burden (%)	90.6 ± 8.1	91.6 ± 7.1	91.3 ± 10.0	0.72
**Pacing parameters at implantation**				
Capture threshold (V/0.4 ms)	1.5 ± 0.4	0.8 ± 0.2	0.6 ± 0.2	<0.01^#^
R-wave amplitude (mV)	5.7 ± 2.6	11.2 ± 3.0	11.5 ± 3.4	<0.01^*^
Impedance (Ω)	664.1 ± 76.6	696.2 ± 124.7	686.3 ± 110.8	0.76
**Parameters at follow-up**				
Capture threshold (V/0.4 ms)	1.7 ± 0.8	0.8 ± 0.1	0.6 ± 0.2	<0.01^#^
R-wave amplitude (mV)	5.6 ± 2.0	11.0 ± 2.2	11.8 ± 3.9	<0.01^*^
Impedance (Ω)	472.8 ± 49.8	507.6 ± 72.3	521.0 ± 78.2	0.20
**Echocardiography at follow-up**				
LVEF (%)	55.8 ± 3.9	54.9 ± 6.7	48.9 ± 9.1	0.02^&^
LVEDD (mm)	49.2 ± 3.3	49.7 ± 5.6	55.0 ± 7.7	0.03^&^
**NYHA functional class at follow-up**				
I	3 (30.0%)	5 (25.0%)	5 (16.7%)	0.61
II	6 (60.0%)	12 (60.0%)	16 (53.3%)	0.87
III	1 (10.0%)	2 (10.0%)	6 (20.0%)	0.55
IV	0 (0.0%)	1 (5.0%)	3 (10.0%)	0.65

*HBP, His bundle pacing; LBBP, left bundle branch pacing; LVEDD, left ventricular end-diastolic diameter; LVEF, left ventricular ejection fraction; NYHA, New York Heart Association; RVP, right ventricular pacing*.

* *P < 0.05 between HBP group vs. LBBP group and RVP group*.

† *P < 0.05 between RVP group vs. HBP group and LBBP group*.

# *P < 0.05 between each group*.

& *P < 0.05 between RVP group vs. LBBP group. Corrected P-value for RVP group vs. HBP group in follow-up LVEF was 0.16; Corrected P-value for RVP group vs. HBP group in follow-up LVEDD was 0.17*.

### Follow-Up Outcomes

The mean follow-up duration after pacemaker implantation was 15.0 ± 9.1 months. The pacing percentages were similar among the three groups (HBP group vs. LBBP group vs. RVP group: 90.6 ± 8.1% vs. 91.6 ± 7.1% vs. 91.3 ± 10.0%, *p* = 0.72). As shown in Table 3, the HBP group still had the highest capture threshold and the lowest R-wave amplitude among groups. For echocardiographic measurements, the LBBP group had significantly higher LVEF (54.9 ± 6.7% vs. 48.9 ± 9.1%, *p* < 0.05) and significantly shorter LVEDD (49.7 ± 5.6 mm vs. 55.0 ± 7.7 mm, *p* < 0.05) than the RVP group. The HBP group had trends of higher LVEF (*p* = 0.16) and lower LVEDD (*p* = 0.17) than the RVP group. The NYHA functional class was improved in all groups during follow-up, and was similar among the three groups.

**Figure 3:**
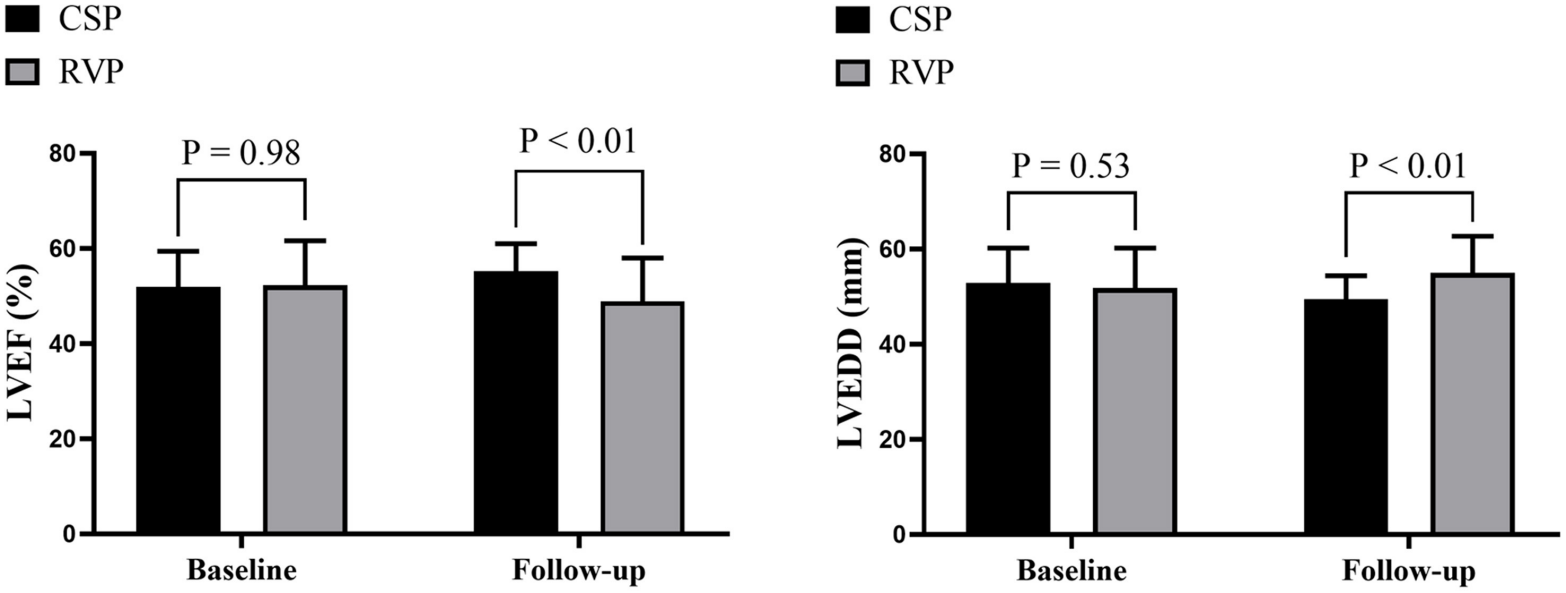
Echocardiographic evaluation between CSP and RVP. CSP, conduction system pacing; LVEDD, left ventricular end-diastolic diameter; LVEF, left ventricular ejection fraction; RVP, right ventricular pacing.

**Figure 4:**
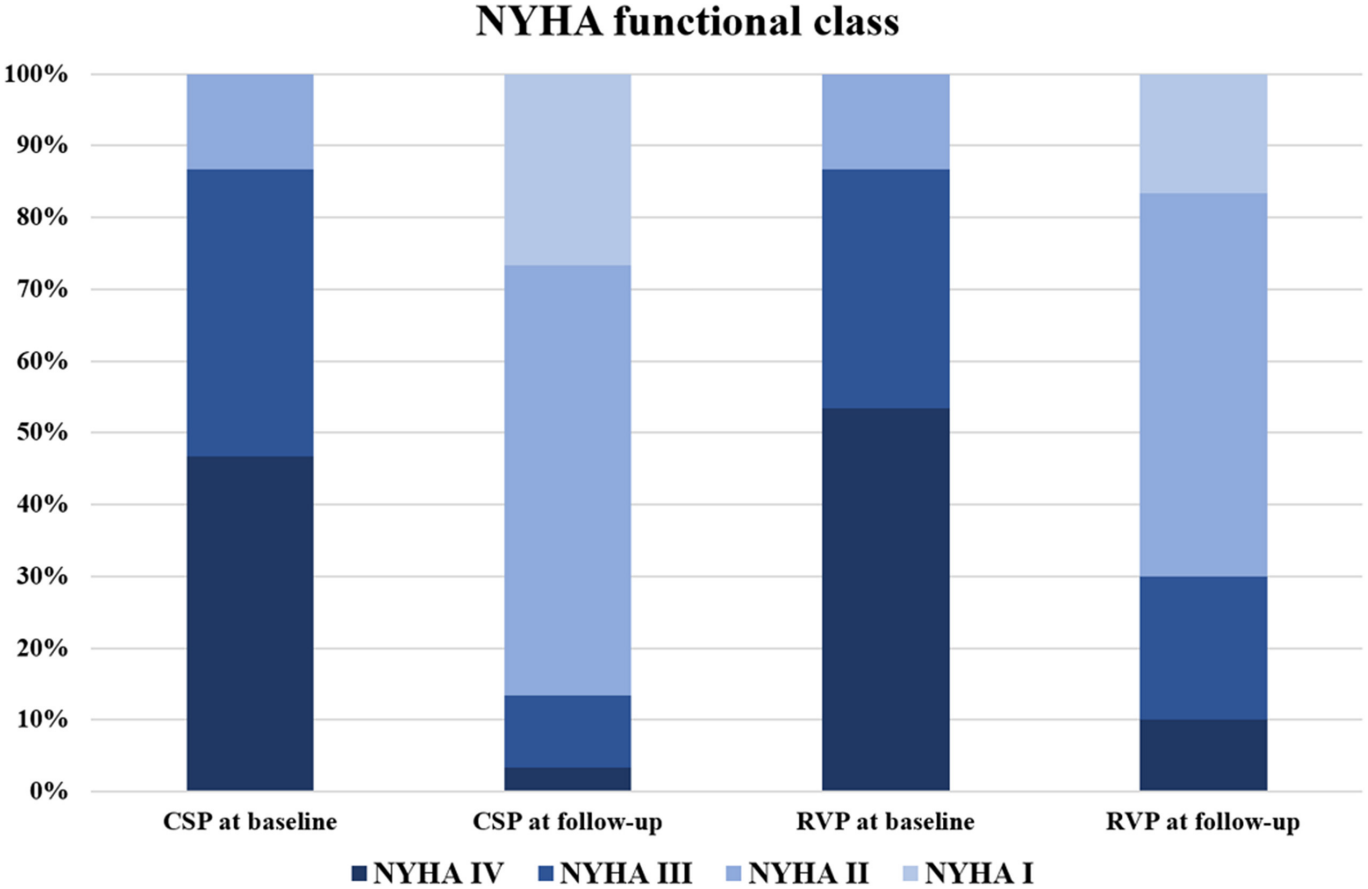
NYHA functional class evaluation between CSP and RVP. CSP at baseline vs. RVP at baseline: *p* = 0.94; CSP at follow-up vs. RVP at follow-up: *p* = 0.47; CSP at baseline vs. CSP at follow-up: *p* < 0.01; RVP at baseline vs. RVP at follow-up: *p* < 0.01. CSP, conduction system pacing; NYHA, New York Heart Association; RVP, right ventricular pacing.

Further analysis between CSP (combining HBP and LBBP) and RVP showed that CSP and RVP had similar baseline echocardiographic parameters, while CSP achieved higher LVEF (55.2 ± 5.8% vs. 48.9 ± 9.1%, *p* < 0.01) and shorter LVEDD (49.5 ± 4.9 mm vs. 55.0 ± 7.7 mm, *p* < 0.01) compared with RVP during follow-up (Figure 3). The NYHA functional class were improved in both types of the pacing modalities during follow-up (Figure 4).

One patient in the HBP group had a capture threshold increase of >1 V/0.4 ms (from 1.6 V/0.4 ms to 3.0 V/0.4 ms) during the 6-month follow-up, while no other procedure-related complications were observed in the other two groups during follow-up. In the RVP group, four patients died: three deaths were due to progressive HF and one death was due to non-cardiac reasons. In the LBBP group, one patient died because of non-cardiac reasons. No death was observed in the HBP group. Three patients in the RVP group required hospitalization for HF; no patients in the other two groups needed hospitalization.

## Discussion

In this study, we evaluated the feasibility of CSP in patients who developed AVB following TAVR, and compared its outcomes with traditional RVP. The main findings were shown as follows: (1) CSP was feasible in post-TAVR patients; (2) CSP obtained a narrower paced QRS duration during the procedure and achieved better cardiac structure and function during follow-up than RVP; and (3) LBBP had higher implant success rate and better pacing parameters than HBP. To the best of our knowledge, this is the first study to directly compare the CSP with RVP in patients who developed AVB following TAVR.

### Conduction Disorders Following TAVR

TAVR is an effective treatment for patients with severe aortic stenosis at moderate-to-high surgical risk. However, the incidence of high-degree AVB or complete AVB is still relatively high because of direct mechanical compression of the artificial valve or perivalvular inflammation or edema caused by the TAVR procedure (11). The incidence of postoperative conduction system block requiring pacemaker implantation was reported in the range of 4.2–17.2% in previous studies (12–14). A long-term follow-up study showed that more than half of the post-TAVR patients with pacemaker implantation had a high percentage of ventricular pacing (15). In these patients, non-physiological pacing modality may offset the therapeutic effect after TAVR and impair the cardiac function. In addition, patients with severe aortic stenosis usually have left ventricular dysfunction; RVP in these patients may aggravate the cardiac dysfunction and lead to poor clinical outcomes. Previous studies have shown that patients with RVP implantation following TAVR had a significantly increased overall mortality compared with patients without pacemaker implantation (16).

### CSP Implantation in Post-TAVR Patients

HBP directly actives the native cardiac conduction system, and is considered as a physiological pacing modality. However, several limitations restrict its wide application including high and unstable capture threshold, low R-wave amplitude, and high lead dislodgement rate (17, 18). In addition, since the lesion site of the conduction system caused by the TAVR procedure is usually located at the distal part of the His–Purkinje system, pacing the HB is difficult to cross the lesion site, resulting in a low implant success rate (19). Previous studies have shown that the implant success rate of HBP in post-TAVR patients is ~50–63% (3, 4).

LBBP can achieve better pacing parameters and similar therapeutic effects by pacing the LBB conduction system, and is considered as an alternative CSP modality (20). In addition, LBBP captures the distal part of the conduction system and can more easily cross the block site, overcoming some limitations in application of HBP in post-TAVR patients (3). As shown in this study, LBBP achieved higher implant success rate, similar paced QRS duration, and more satisfactory pacing parameters than HBP. All these suggest that LBBP is more suitable than HBP as the primary treatment option for patients who need pacing therapy after TAVR.

### Therapeutic Effects of Different Pacing Modalities

In this study, in addition to evaluating the feasibility of CSP implantation in post-TAVR populations, we also compared the echocardiographic measurements of CSP with traditional RVP. The results showed that for patients with pacemaker implantation after TAVR, CSP achieved better LVEF and LVEDD compared with RVP. To the best of our knowledge, this is the first study to compare the echocardiographic measurements of different pacing modalities in post-TAVR patients. However, due to the low incidence of the clinical endpoints in this study, we were unable to further evaluate whether this echocardiographic benefit could be translated into better long-term clinical outcomes.

### Limitations

Our study has some limitations. First, this is a non-randomized, single-center study with a relatively small sample size. The sample size in the HBP group was small, resulting in insufficient statistical power to compare the difference in echocardiographic measurements compared to the other two groups. In addition, due to the small sample size, we were unable to identify the specific subgroup in the RVP group that was responsible for the worse echocardiographic measurements, nor to evaluate the risk factors of lead septal perforation in the elderly LBBP population (21). Multicenter randomized studies with larger sample size are needed to further confirm these conclusions. Second, the enrolled patients were all implanted with the same valve type, other valve types, especially balloon-expandable valves, may cause different types of injury to the conduction system, which may lead to different physiological characteristics and clinical outcomes. Finally, the low incidence of clinical endpoints made it difficult to compare the differences of clinical endpoints between groups. Further studies with larger sample size and longer follow-up duration are needed to evaluate the long-term clinical outcomes.

## Conclusions

CSP achieved shorter paced QRS duration and better cardiac structure and function than RVP in patients who developed AVB following TAVR. Furthermore, LBBP had higher implant success rate and better pacing parameters than HBP.

## Data Availability Statement

The raw data supporting the conclusions of this article will be made available by the authors, without undue reservation.

## Ethics Statement

The studies involving human participants were reviewed and approved by this study was approved by the Ethics Committee of Fuwai Hospital, and all patients provided written consent for participation. The patients/participants provided their written informed consent to participate in this study.

## Author Contributions

WH and SZ contributed to the study conception and design. H-XN, MG, XC, and CC performed pacemaker implantation. H-XN, XL, MG, and MC performed data collection and analysis. The first draft of the manuscript was written by H-XN and XL, and all authors commented on previous versions of the manuscript. All authors contributed to the article and approved the submitted version.

## Funding

This work was supported by the National Natural Science Foundation of China (Grant Number: 82070349), the Peking Union Medical College Youth Fund and Fundamental Research Funds for the Central Universities (Grant Numbers: 3332019047 and 2017320006), CAMS Innovation Fund for Medical Sciences (CIFMS, Grant Number: 2021-I2M-C&T-B-028), and the Innovation Funds for Graduate Students of Peking Union Medical College (Grant Number: 2019-1002-33).

## Conflict of Interest

The authors declare that the research was conducted in the absence of any commercial or financial relationships that could be construed as a potential conflict of interest.

## Publisher's Note

All claims expressed in this article are solely those of the authors and do not necessarily represent those of their affiliated organizations, or those of the publisher, the editors and the reviewers. Any product that may be evaluated in this article, or claim that may be made by its manufacturer, is not guaranteed or endorsed by the publisher.
